# Nonsteroidal anti‐inflammatory drugs prevent gastric cancer associated with the use of proton pump inhibitors after *Helicobacter pylori* eradication

**DOI:** 10.1002/jgh3.12583

**Published:** 2021-06-05

**Authors:** Junya Arai, Ryota Niikura, Yoku Hayakawa, Hiroki Sato, Takuya Kawahara, Tetsuro Honda, Kenkei Hasatani, Naohiro Yoshida, Tsutomu Nishida, Tetsuya Sumiyoshi, Shu Kiyotoki, Takashi Ikeya, Masahiro Arai, Nobumi Suzuki, Yosuke Tsuji, Atsuo Yamada, Kazuhiko Koike

**Affiliations:** ^1^ Department of Gastroenterology, Graduate School of Medicine The University of Tokyo Bunkyo City Tokyo Japan; ^2^ Clinical Research Promotion Center The University of Tokyo Hospital Bunkyo City Tokyo Japan; ^3^ Department of Gastroenterology Nagasaki Harbor Medical Center Nagasaki‐shi Nagasaki Japan; ^4^ Department of Gastroenterology Fukui Prefectural Hospital Fukui‐shi Fukui Japan; ^5^ Department of Gastroenterology Ishikawa Prefectural Central Hospital Kanazawa‐shi Ishikawa Japan; ^6^ Department of Gastroenterology Toyonaka Municipal Hospital Toyonaka‐shi Osaka Japan; ^7^ Department of Gastroenterology Tonan Hospital Sapporo‐shi Hokkaido Japan; ^8^ Department of Gastroenterology Shuto General Hospital Yanai‐shi Yamaguchi Japan; ^9^ Department of Gastroenterology St. Luke's International Hospital Chuo‐ku Tokyo Japan; ^10^ Department of Gastroenterology Nerima Hikarigaoka Hospital Nerima‐ku Tokyo Japan; ^11^ Department of Gastroenterological Endoscopy Tokyo Medical University Shinjuku‐ku Tokyo Japan

**Keywords:** COX2 inhibitors, gastric cancer, *Helicobacter pylori*, NSAIDs, proton pump inhibitor

## Abstract

**Background and Aim:**

Proton pump inhibitors (PPIs) are a potential cause of gastric carcinogenesis after *Helicobacter pylori* eradication. Thus, appropriate management including chemoprevention is required. The aim of this study was to evaluate the association between nonsteroidal anti‐inflammatory drugs (NSAIDs) and the incidence of post‐eradication gastric cancer in PPI users.

**Methods:**

A multicenter retrospective cohort study was conducted. Patients who used a PPI (≥30 days) after *H. pylori* eradication between 2014 and 2019 were analyzed in nine hospital databases. Gastric cancer incidence was a primary outcome, and their association with NSAIDs use and clinical factors was evaluated. Hazard ratios were adjusted by age, sex, smoking, and Charlson Comorbidity Index.

**Results:**

During the mean follow‐up period of 2.38 years, 1.13% (31/2431) of all patients developed gastric cancer. The cumulative incidence of gastric cancer in PPI users was 0.25% at 1 year, 0.51% at 3 years, and 1.09% at 5 years in the NSAID users and 0.89% at 1 year, 2.32% at 3 years, and 3.61% at 5 years in nonusers. NSAIDs were associated with a lower gastric cancer risk (adjusted hazard ratio = 0.28, *P* = 0.005). No gastric cancer was observed in the cyclooxygenase‐2 inhibitor users (*n* = 256). NSAID use with high dose and long duration was significantly associated with a lower incidence of gastric cancer.

**Conclusion:**

NSAIDs were associated with a 60% decrease in the gastric cancer incidence in *H. pylori*‐eradicated PPI users, with dose and duration response effects. NSAIDs may be effective for chemoprevention against PPI‐related gastric cancer.

## Graphical abstract

Nonsteroidal anti‐inflammatory drugs (NSAIDs) were associated with a 60% decrease in the gastric cancer incidence in *Helicobacter pylori*‐eradicated proton pump inhibitor (PPI) users, with dose and duration response effects. NSAIDs may be effective for chemoprevention against PPI‐related gastric cancer.

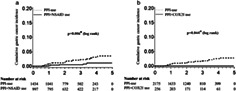

## Introduction

Gastric cancer is the third leading cause of cancer‐related mortality worldwide.^1^ *Helicobacter pylori* is the most important carcinogen for gastric cancer,^2^ and *H. pylori* eradication reduces gastric cancer risk by 47%. However, the annual incidence rate of post‐eradication gastric cancer is approximately 1.4%,^3^ and understanding its pathogenesis and establishing a novel preventive approach remain to be required.

Previous reports revealed that proton pump inhibitor (PPI) use was associated with a 2.4‐fold increase in the risk of *H. pylori* post‐eradication gastric cancer (post‐eradication gastric cancer).^4^, ^5^ Another meta‐analysis also supported a possible association between long‐term use of PPIs and the risk of gastric cancer.^6^ Although PPIs may be a potential risk factor for post‐eradication gastric cancer, they are among the most commonly used drug groups in the world, and millions of patients need them for peptic ulcers and reflux diseases. Identification of populations at high risk of post‐eradication gastric cancer and drugs effective for chemoprevention for the high‐risk population would be beneficial for appropriate management of *H. pylori*‐eradicated PPI users.

Nonsteroidal anti‐inflammatory drugs (NSAIDs) are potential chemopreventive drugs for gastric cancer. NSAIDs were associated with a 58% decrease in gastric cancer incidence in a country with a high rate of *H. pylori* infection,^7^ and a similar effect was found in a country with a low rate.^8^ Therefore, we hypothesized that NSAIDs may be preventive for gastric cancer development even in *H. pylori*‐eradicated PPI users. However, NSAIDs increase gastrointestinal (GI) bleeding risk and potentially cardiovascular risk. The balance between the benefits and risks of NSAID use must be considered.

To address these issues, we performed a multicenter retrospective cohort study of patients who used PPIs after eradication and evaluated the associations between NSAID use and both gastric cancer development and adverse events, including upper GI bleeding and cardiovascular diseases. Furthermore, we evaluated the association between gastric cancer and patients' genotype of CYP2C19, which is involved in PPI metabolism. The findings may be useful for identification of optimal PPI users who require additional chemopreventive intervention.

## Methods

### Study design, setting, and participants

We performed a retrospective cohort study using the diagnostic procedure combination databases from nine hospitals between April 2014 and March 2019. The combined database provided records from all inpatients and outpatients of the University of Tokyo Hospital, Shuto General Hospital, Fukui Prefectural Hospital, Nerima Hikarigaoka Hospital, St. Leuk's International Hospital, Toyonaka Municipal Hospital, Ishikawa Prefectural Central Hospital, and Nagasaki Minato Medical Center and from inpatients of Tonan Hospital. The database included diagnosis, comorbidities, and adverse events using the International Classification of Diseases, tenth revision (ICD‐10), and drugs and procedures coded according to the original Japanese code.

From the database, we extracted the data of patients who used a 7‐day course of clarithromycin‐based or metronidazole‐based triple therapy for *H. pylori* infection between April 2014 and March 2019 using the drug codes (Table S1). We excluded patients who had used PPIs for less than 30 days and developed gastric cancer within half a year after *H. pylori* eradication. Patients who have history of gastrectomy before *H. pylori* eradication were also excluded. The follow‐up period was from the date of eradication drug use to the final visit. The end of follow‐up was March 2019, and loss to follow‐up was defined as the date of the final visit. The study was approved by the institutional review boards of the University of Tokyo Hospital (no. 2019161NI).

### Outcomes and variables

The primary outcome was the development of gastric cancer, as defined by the ICD‐10 codes (C160, C161, C162, C163, C164, C165, C166, C168, and C169) or procedure codes for endoscopic and surgical resection (K6531, K6532, K6533, K6534, K654‐2, K654‐31, K654‐32, K6551, K6552, K655‐21, K655‐22, K655‐41, K655‐42, K655‐51, K655‐52, K656, K656‐2, K656‐2, K6571, K6572, K657‐21, and K657‐22).

The secondary outcomes were upper GI bleeding and cardiovascular diseases, including cerebrovascular diseases and ischemic heart diseases. Upper GI bleeding was defined as performing endoscopic hemostasis for GI bleeding, with a procedure code of K654. Cerebrovascular disease was defined by ICD‐10 codes G450–469, H340, I600–639, I64, and I650–699, and ischemic heart disease was defined by ICD‐10 codes I210–I229 and I252.

We evaluated the following clinical factors: age, sex, smoking, comorbidities, and mediation use. Age was categorized into two groups: >70 and ≤70 years. The following comorbidities were included based on ICD codes: atrial fibrillation, acquired immunodeficiency syndrome, arterial thrombosis, carotid disease, cerebrovascular disease, chronic heart failure, chronic kidney disease (stage 5 or lower), dementia, diabetes mellitus with or without complications, deep vein thrombosis, hemiplegia, dyslipidemia, ischemic heart diseases, liver disorder (mild/severe), malignancy with or without metastasis, pulmonary embolism, peripheral vascular disease, pulmonary disease, rheumatic disease, transient ischemic attack, peptic ulcer disease, unstable angina disease, and valvular disease. The Charlson Comorbidity Index was calculated using these data.^9^ The details of ICD‐10 codes are shown in Table S2.

Usage of NSAIDs, including COX2 (cyclooxygenase‐2) inhibitors, aspirin, metformin, and statins with other lipid‐lowering agents (fibrates and others), was assessed. NSAIDs were defined as loxoprofen, sulindac, diclofenac, flurbiprofen, ibuprofen, indomethacin, ketoprofen, oxaprozin, naproxen, mefenamic acid, flufenamate aluminum, acemetacin, proglumetacin maleate, mofezolac, pranoprofen, tiaprofenic acid, zaltoprofen, tiamide hydrochloride, etodolac, meloxicam, nabumetone, zaltoprofen, lornoxicam, and piroxicam, including COX2 inhibitors (celecoxib). Statins included pitavastatin, simvastatin, pravastatin, fluvastatin, atorvastatin, and rosuvastatin. Rosuvastatin, pitavastatin, and atorvastatin were defined as strong statins. Fibrates included fenofibrate, bezafibrate, clinofibrate, and clofibrate. The duration of NSAID use was categorized as short‐term (<30 days) or long‐term (≥30 days). The NSAID dose was categorized as low (one tablet) or high (two tablets or more). The details of the medication codes of the medications are shown in Table S1.

### Genotyping

Genotyping of the CYP2C19 wild‐type gene and two mutated alleles (mutations CYP2C19m1 in exon 5 and CYP2C19m2 in exon 4) was performed using polymerase chain reaction (PCR) restriction fragment length polymorphism (TaqMan Sample‐to‐SNP kit) for patients who consented to the examination. The patients were categorized into three groups: extensive metabolizers (*1/*1), intermediate metabolizers (*1/*2 and *1/*3), and poor metabolizers (*2/*2, *2/*3, and *3/*3).

### Statistical analysis

The primary endpoint, gastric cancer development, was censored at the date of the final visit. The Kaplan–Meier method was used to calculate the cumulative probability of gastric cancer development at 5 years. Univariate and multivariate Cox models were used to estimate hazard ratios (HRs) and 95% confidence intervals (CIs); the multivariate Cox proportional hazards models were adjusted for age and sex. Missing data for sex (*n* = 1355) and age (*n* = 1355) were imputed using the fitted values from general linear models for comorbidities.

We also estimated the HRs for gastric cancer development according to NSAID duration and dose using other Cox models. In addition, we performed survival analyses using Cox models to evaluate the associations between NSAID use and the secondary endpoints (GI bleeding and cardiovascular disease). We calculated the posterior probabilities of gastric cancer using the Bayesian model in patients who underwent CYP2C19 genotyping.

A *P* value <0.05 was considered statistically significant, and HRs with 95% CIs were determined. All statistical analyses were performed using SAS software v. 9.4 (SAS Institute, Cary, NC, USA).

### Patient and public involvement

Patients and the public were neither involved in the study design nor the recruitment.

This study received ethics approval from the institutional review boards of the University of Tokyo (ID: 2058‐(2)).

## Results

### Patient characteristics

**Figure 1:**
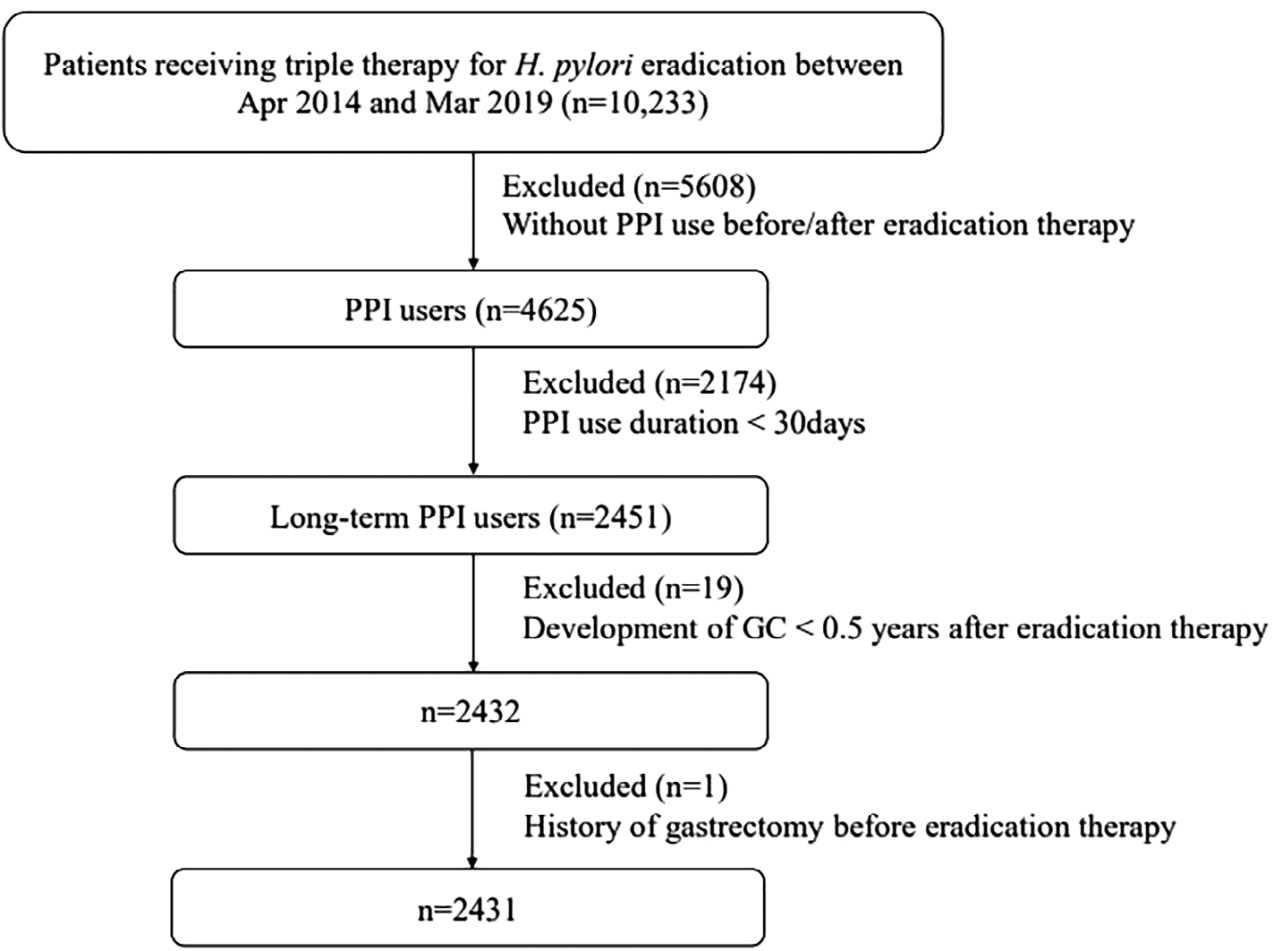
Patient selection flow diagram. GC, gastric cancer; *H. pylori*, *Helicobacter pylori*; PPI, proton pump inhibitor.

A total of 10 233 patients received *H. pylori* eradication therapy. After excluding 7802 patients, 2431 *H. pylori*‐eradiated PPI users were analyzed (Fig. 1). Of these 2431 patients, the mean age was 68.12 years, 58.46% (*n* = 629) were male (missing data *n* = 1355), and 41.01% used NSAIDs. High‐ and low‐dose and short‐term and long‐term NSAID users made up 2.71, 38.30, 23.36, and 17.65% of the study population, respectively. The percentages of aspirin, statin, strong statin, fibrate, other lipid lowering agent, and metformin users were 17.03, 26.00, 22.75, 2.38, 3.50, and 6.38%, respectively. The baseline characteristics of the cohort are shown in Table 1.

**Table 1:** Patient characteristics of long‐term proton pump inhibitor (PPI) users

Characteristic	Number of patients (%)
Male (missing data *n* = 1355)	629 (58.46)
Age > 70 years (missing data *n* = 1355)	550 (51.12)
Follow‐up years	2.38 ± 1.45 years
Smoking	427 (17.56)
Comorbidities	
Atrial fibrillation	23 (0.95)
AIDS	0 (0.00)
Arterial thrombosis	18 (0.74)
Carotid disease	5 (0.21)
Cerebrovascular disease	74 (3.04)
Chronic heart failure	135 (5.55)
Chronic kidney disease < stage 5	42 (1.73)
Chronic kidney disease stage 5	4 (0.16)
Dementia	15 (0.62)
DM without complications	231 (9.50)
DM with complications	83 (3.41)
Deep vein thrombosis	17 (0.70)
Hemiplegia	1 (0.04)
Hypertension	332 (13.66)
Dyslipidemia	210 (8.64)
Ischemic heart diseases	23 (0.95)
Liver disorder (mild level)	99 (4.07)
Liver disorder (severe level)	14 (0.58)
Malignancy without metastasis	166 (6.83)
Malignancy with metastasis	36 (1.48)
Pulmonary embolism	0 (0.00)
Peripheral vascular diseases	38 (1.56)
Pulmonary disease	82 (3.37)
Rheumatic diseases	27 (1.11)
Transient ischemic attack	2 (0.08)
Peptic ulcer diseases	266 (10.94)
Unstable angina diseases	122 (5.02)
Valvular diseases	26 (1.07)
Charlson Comorbidities Index	0.72 ± 1.57
Medications	
Aspirin	414 (17.03)
NSAIDs	997 (41.01)
COX2I (only celecoxib)	256 (10.53)
Statin (total)	632 (26.00)
Strong statin	553 (22.75)
Fibrate	57 (2.34)
Other lipid lowering	85 (3.50)
Metformin	155 (6.38)

AIDS, acquired immunodeficiency syndrome; DM, diabetes mellitus; NSAIDs, nonsteroidal anti‐inflammatory drugs; COX2I, cyclooxygenase‐2 inhibitor.

PPIs were principally used for gastroesophageal reflux related symptoms. In addition, PPI users had histories of 14.64% of gastroesophageal reflux disease, 8.68% of cardiovascular diseases, and 10.94% of previous GI bleeding. NSAIDs were principally used for orthopedic disease related pain. In addition, NSAID users had histories of 10.83% of cardiovascular disease, 2.01% of rheumatic diseases, and 2.51% of orthopedic disease including osteoarthritis, spinal disc herniation, and spinal stenosis.

### Incidence of gastric cancer and the factors associated with gastric cancer in the long‐term PPI users

**Figure 2:**
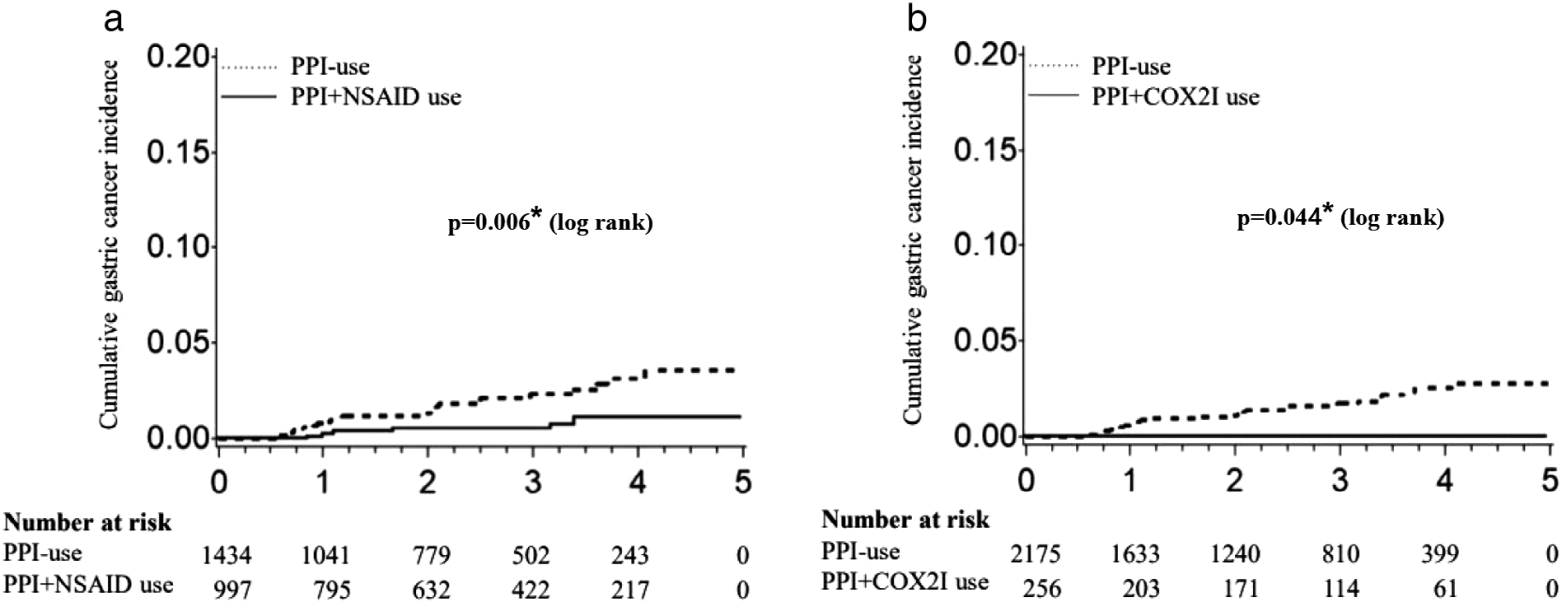
Cumulative incidence of gastric cancer in (a) PPI + NSAID users *versus* PPI‐users and (b) PPI + COX2I users *versus* PPI‐users. Survival analysis was performed using the Kaplan–Meier method and log‐rank test. COX2I, selective cyclooxygenase‐2 inhibitors; NSAID, nonsteroidal anti‐inflammatory drug; PPI, proton pump inhibitor.

During the mean follow‐up period of 2.38 years, 31 patients (1.13%) developed gastric cancer (2 in the cardia, 14 in the non‐cardia region; data were not available for 15 patients). The cumulative incidence of gastric cancer was 0.24% at 1 year, 0.52% at 3 years, and 1.09% at 5 years in the patients using NSAIDs and PPI, and 0.89% at 1 year, 2.32% at 3 years, and 3.61% at 5 years in the patients using PPI alone (Fig. 2a). NSAID use was significantly associated with decreased gastric cancer incidence (*P* = 0.006, log‐rank test).

The cumulative incidence of gastric cancer was 0% at 1, 3, and 5 years in the patients who used COX2 inhibitors with PPI, and 0.69% at 1 year, 1.72% at 3 years, and 2.81% at 5 years in the patients who used PPI but not COX2 inhibitors (Fig. 2b). The use of COX2 inhibitors was significantly associated with a decreased gastric cancer incidence (*P* = 0.044, log‐rank test).

**Figure 3:**
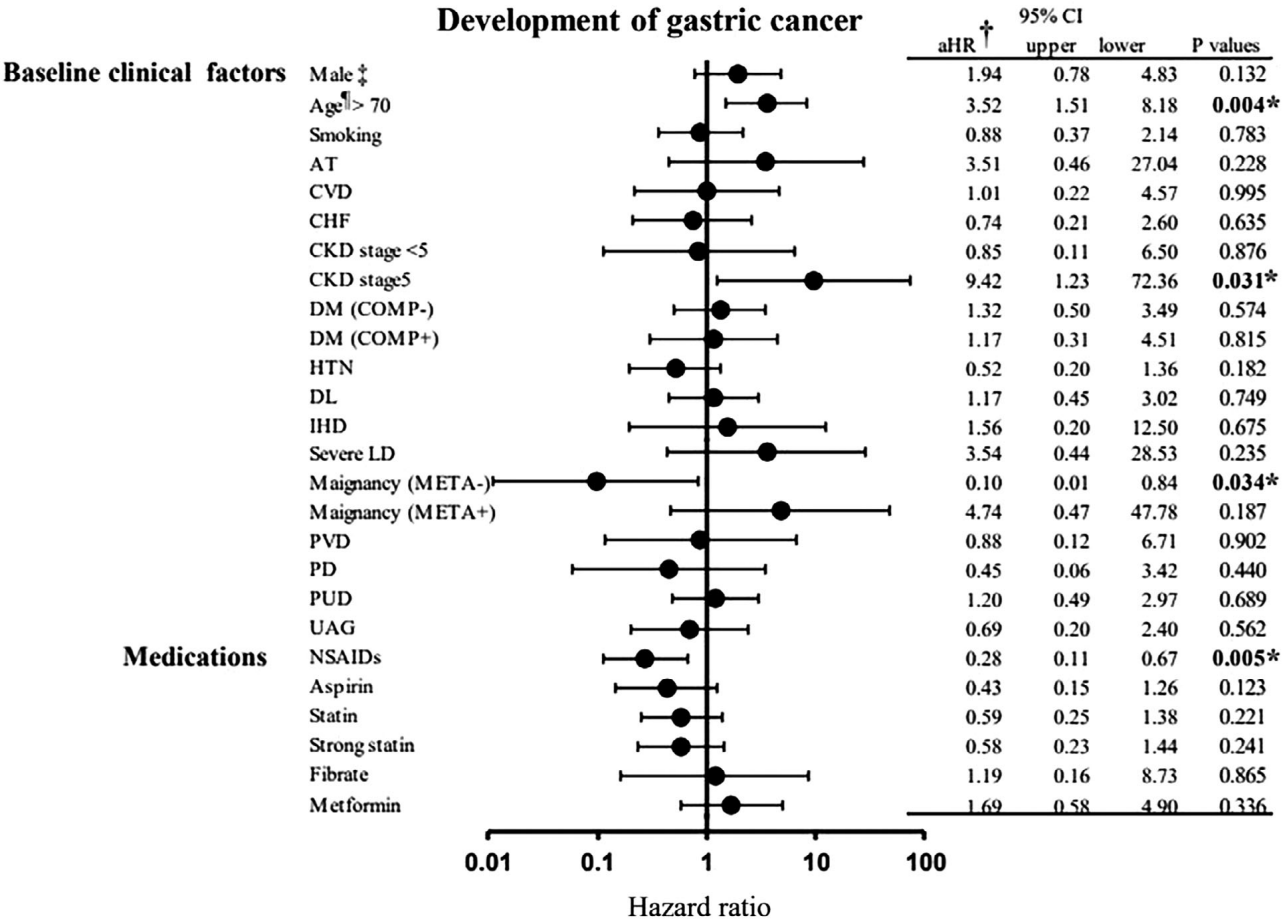
Hazard ratios for development of gastric cancer according to clinical factors in long‐term PPI users. †HR adjusted for age >70 years, sex, smoking, and Charlson Comorbidity Index. ‡Male was defined as the sex predicted value >0.5, using the fitted values from general linear models for comorbidities. §Age was including the age predicted values, using the fitted values from general linear models for comorbidities. aHR, adjusted hazard ratio; AT, arterial thrombosis; CHF, chronic heart failure; CI, confidence interval; CKD, chronic kidney disease; COMP, complications; CVD, cerebrovascular disease; DL, dyslipidemia; DM, diabetes mellitus; HTN, hypertension; LD, liver disorder; META, metastasis; NSAIDs, nonsteroidal anti‐inflammatory drugs; PD, pulmonary disease; PUD, peptic ulcer disease; PVD, peripheral vascular disease; UAG, unstable angina disease.

The results of a Cox model evaluating factors associated with gastric cancer are shown in Table 2 and Figure 3. In the analyzed *H. pylori*‐eradicated PPI users, NSAID use was associated with a lower incidence of gastric cancer compared with non‐use (adjusted HR [aHR] 0.28, 95% CI 0.11–0.67, *P* = 0.005). The use of other drugs including aspirin, statin, fibrate, and metformin was not significantly associated with gastric cancer incidence. Analysis of other factors showed that a higher incidence of gastric cancer was associated with age >70 years (aHR 3.52, 95% CI 1.51–8.18, *P* = 0.004), chronic kidney disease stage 5 (aHR 9.42, 95% CI 1.23–72.36, *P* = 0.041), and malignancy without metastasis (aHR 0.10, 95% CI 0.01–0.84, *P* = 0.034).

**Table 2:** Factors of medications associated with gastric cancer development in long‐term proton pump inhibitor (PPI) users

Factor	Gastric cancer, *n* = 31	Non‐gastric cancer, *n* = 2400	Crude HR (95% CI)	Adjusted HR† (95% CI)	*P* value
PPI‐use	25 (1.74)	1409 (98.26)	1	1	
PPI + NSAID use	6 (0.60)	991 (99.40)	0.30 (0.13–0.74)	0.28 (0.11–0.67)	0.005*
PPI‐use	31 (1.43)	2144 (98.57)	1	1	
PPI + COX2I use	0 (0.00)	256 (100.00)	NA	NA	—
PPI‐use	27 (1.34)	1990 (98.66)	1	1	
PPI + aspirin use	4 (0.97)	410 (99.03)	0.67 (0.23–1.90)	0.43 (0.15–1.26)	0.123
PPI‐use	24 (1.33)	1775 (98.67)	1	1	
PPI + statin use	7 (1.11)	625 (98.89)	0.74 (0.32–1.72)	0.59 (0.25–1.38)	0.221
PPI‐use	25 (1.33)	1853 (98.67)	1	1	
PPI + strong statin use	6 (1.08)	547 (98.92)	0.75 (0.31–1.82)	0.58 (0.23–1.44)	0.241
PPI‐use	30 (1.26)	2344 (98.74)	1	1	
PPI + fibrate use	1 (1.75)	56 (98.25)	1.32 (0.18–9.67)	1.19 (0.16–8.73)	0.865
PPI‐use	31 (1.32)	2315 (98.68)	1	1	
Use of PPI + other lipid‐lowering agents	0 (0.00)	85 (100.00)	NA	NA	—
PPI‐use	27 (1.19)	2249 (98.81)	1	1	
PPI + Metformin use	4 (2.58)	151 (97.42)	1.97 (0.69–5.63)	1.69 (0.58–4.90)	0.336

† HR adjusted for age > 70 years, sex, smoking, and Charlson Comorbidity Index.

CI, confidence interval; COX2I, cyclooxygenase‐2 inhibitor; HR, hazard ratio; NA, not applicable; NSAID, nonsteroidal anti‐inflammatory drug.

### Sub‐analysis of the association between the dose or duration of NSAID use and gastric cancer in long‐term PPI users

Of the 931 patients using low‐dose NSAIDs, six patients developed gastric cancer (0.64%). None of the 66 high‐dose NSAID users developed gastric cancer. The associations between dose or duration of NSAID use and gastric cancer incidence were assessed by using a Cox model (Table 3). The aHR for low‐dose NSAID use was 0.29 (95% CI 0.12–0.71) compared with non‐use. In terms of duration of NSAID use, aHRs for short‐term (<30 days) and long‐term (≥30 days) use were 0.44 (95% CI 0.15–1.27) and 0.16 (95% CI 0.037–0.66) compared with the non‐use group.

**Table 3:** Association between the dose/duration of nonsteroidal anti‐inflammatory drug (NSAID) use and gastric cancer incidence in long‐term proton pump inhibitor (PPI) users

Factor	Gastric cancer, *n* = 31	Non‐gastric cancer, *n* = 2399	Crude HR (95% CI)	Adjusted HR† (95% CI)	*P* value
Dose					
PPI‐use	25 (1.74)	1409 (98.26)	1	1	
PPI + LD‐NSAID use	6 (0.64)	925 (99.36)	0.33 (0.13 to 0.80)	0.29 (0.12 to 0.71)	0.007*
PPI + HD‐NSAID use	0 (0.00)	66 (100.00)	Not applicable	Not applicable	—
Duration					
PPI‐use	25 (1.74)	1409 (98.26)	1	1	
PPI + short NSAID use	4 (0.93)	425 (99.07)	0.49 (0.17 to 1.42)	0.44 (0.15 to 1.27)	0.128
PPI + long NSAID use	2 (0.35)	566 (99.65)	0.17 (0.041 to 0.72)	0.16 (0.037 to 0.66)	0.012*

† HR adjusted for age > 70 years, sex, smoking, and Charlson Comorbidity Index.

CI, confidence interval; HD, high dose; HR, hazard ratio; LD, low dose; NSAID, nonsteroidal anti‐inflammatory drug.

### Incidences of cardiovascular diseases and upper GI bleeding in long‐term PPI users

**Figure 4:**
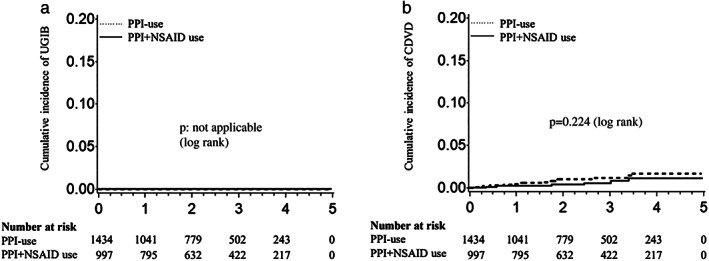
Cumulative incidence of adverse events in PPI + NSAID users *versus* PPI‐users: (a) UGIB and (b) CDVD. Survival analysis was performed using the Kaplan–Meier method and log‐rank test. CDVD, cardiovascular disease; NSAIDs, nonsteroidal anti‐inflammatory drugs; PPI, proton pump inhibitor; UGIB, upper gastrointestinal bleeding.

Figure 4 shows a Kaplan–Meier plot for the incidences of adverse events including upper GI bleeding and cardiovascular diseases in the NSAID users and nonusers. No patients developed upper GI bleeding in either group (Fig. 4a). Twenty patients (0.82%) developed cardiovascular events. The cumulative incidence of cardiovascular events was 0.22% at 1 year, 0.55% at 3 years, and 1.10% at 5 years in the NSAID use group and 0.49% at 1 year, 1.21% at 3 years, and 1.70% at 5 years in the non‐use group (Fig. 4b). No significant difference in the incidence of cardiovascular events was observed between the groups (*P* = 0.224, log‐rank test).

### CYP2C19 genotyping

In order to investigate the effects by PPI metabolism in cancer incidence, CYP2C19 genotype was measured in a total of 199 patients. Of these patients, 78.9% were aged >70 years, 72.4% were men. Genotyping results indicated that 77, 90, and 32 patients were CYP2C19 extensive, intermediate, and poor metabolizers, respectively. A total of five patients had gastric cancer. The risk of gastric cancer (aHR 2.47) in the CYP2C19 non‐extensive metabolizers, that is, the intermediate and poor metabolizers, was elevated compared with that in extensive metabolizers; however, this difference was not statistically significant (Tables S3 and S4). Nevertheless, a Bayesian model showed the potential risk of gastric cancer in the non‐extensive metabolizers. Combined with the uninformative prior distribution and data, the posterior probabilities of a greater hazard in CYP2C19 intermediate and poor metabolizers than in extensive metabolizers were 79.8 and 87.6%, respectively (Figure S2), suggesting that impaired CYP2C19 metabolizers may encounter prolonged effects by PPI, which potentially further increase the gastric cancer risk.

## Discussion

In this multicenter cohort study, we found that NSAID use was associated with a 60% decrease in the post‐eradication gastric cancer incidence in PPI users, and dose– and duration–response relationships were confirmed. NSAID use was not associated with increased GI bleeding or cerebrovascular events. In addition, COX2 inhibitors showed relatively higher chemopreventive effects, and we identified CYP2C19 poor metabolizers as a potential high‐risk population for post‐eradication gastric cancer.

*H. pylori* eradication therapy has been widely encouraged for prevention of peptic ulcers, as well as subsequent gastric cancers. However, a significant population in *H. pylori*‐eradicated patients often need the prolonged use of PPIs, due to refractory acid‐reflux symptoms. Given the potential cancer‐promoting effects by PPIs in such patients,^4^, ^5^ there is a clinical dilemma when seeing *H. pylori*‐eradicated PPI users. Our current finding that NSAIDs efficiently prevent gastric cancer development in PPI users would be helpful for solving this problem, that is, additional chemopreventive use of NSAIDs may be considered for a subgroup of *H. pylori*‐eradicated PPI users.

We found that CYP2C19 non‐extensive metabolizers who use PPIs are at high risk of gastric cancer. NSAID use as chemoprevention may be beneficial in these patients following *H. pylori* eradication. In addition, given that past history of gastric cancers and advanced histopathological changes such as gastric atrophy and intestinal metaplasia are known to be a risk factor for post‐eradication gastric cancer, patients with these factors may also be a potential target of chemopreventive approach using NSAIDs.

Since NSAIDs are anti‐inflammatory drug, their anti‐inflammatory effects may contribute to a low incidence of gastric cancer. In addition, several in vitro and in vivo studies suggested that COX2 may play a critical role in proliferation and tumor growth in gastric cancers.^10^, ^11^ Given the strong cancer‐preventing effects by COX2 inhibitors in our cohort and other studies,^12^ COX2 pathway may be central in gastric cancer development at least in a subset of chronic gastritis patients.

Contrary to previous reports,^13^, ^14^, ^15^ NSAID use was not associated with increased upper GI bleeding or cardiovascular events in our study. One explanation for the discrepancy might be that all our patients received *H. pylori* eradication therapy and used PPIs, which theoretically lower the incidence of upper GI bleeding. In our study, the rate of comorbidities associated with a cardiovascular risk, such as carotid, cerebrovascular, peripheral vascular, and ischemic heart diseases, was also fewer (5%) than in previous studies (more than 10%).^14^, ^15^ While this discrepancy might be due to shorter follow‐up period and fewer dosage of NSAIDs in our study, our data suggest that chemopreventive use of NSAIDs may be feasible for *H. pylori*‐eradicated PPI users.

Our study has several strengths. First, to our best knowledge, this was the first study to evaluate the association between NSAID use and the incidence of gastric cancer after *H. pylori* eradication in PPI users. Second, this was a large multicenter cohort study. Nevertheless, our study has several limitations. First, this study was retrospective. Second, our diagnostic procedure combination database lacked data on successful eradication of *H. pylori*. However, the success rate of *H. pylori* eradication was expected to be greater than 90%, based on the primary use of vonoprazan‐based drugs in Japan.^16^ Third, the follow‐up period was relatively short and insufficient to accurately evaluate gastric cancer events. However, we will expand our database to double the number of hospitals and will perform further studies for another 2 years in near future. Lastly, information on some risk factors, including diet, body mass index, and family history, was limited in our database.

In conclusion, NSAIDs were associated with a decreased risk of gastric cancer with dose– and duration–response relationships among PPI users after *H. pylori* eradication, without adverse events. NSAIDs may be a candidate for prevention of PPI‐related gastric cancer.

## Supporting information


**Figure S1.** Cumulative incidence of gastric cancer according to (A) CYP2C19 extensive *vs* non‐extensive metabolizers and (B) CYP2C19 extensive *vs* intermediate *vs* poor metabolizers.
**Figure S2.** Posterior probability of gastric cancer in CYP2C19 intermediate and poor metabolizers using a Bayesian model
**Figure S3.** Cumulative incidence of gastric cancer in (A) PPI+NSAID high‐dose and low‐dose users and PPI‐users, (B) PPI+NSAID long‐term and short‐term users and PPI‐users.
**Table S2.** ICD10 codes of comorbidities.
**Table S3.** Association between CYP2C19 extensive and non‐extensive metabolizers and gastric cancer incidence (*n* = 199).
**Table S4.** Associations of gastric cancer incidence with CYP2C19 extensive, intermediate, and poor metabolizers (*n* = 199).

**Table S1.** Drug codes.
