# Supplementary material for: Nonsteroidal anti‐inflammatory drugs prevent gastric cancer associated with the use of proton pump inhibitors after Helicobacter pylori eradication
Source: JGH Open. 2021 Jun 5;5(7):770–7. doi: 10.1002/jgh3.12583 (PMC8264245; doi:10.1002/jgh3.12583)
Supplement: Supplementary file 1 — Figure S1. Cumulative incidence of gastric cancer according to (A) CYP2C19 extensive vs non‐extensive metabolizers and (B) CYP2C19 extensive vs intermediate vs poor metabolizers. Figure S2. Posterior probability of gastric cancer in CYP2C19 intermediate and poor metabolizers using a Bayesian model Figure S3. Cumulative incidence of gastric cancer in (A) PPI+NSAID high‐dose and low‐dose users and PPI‐users, (B) PPI+NSAID long‐term and short‐term users and PPI‐users. Table S2. ICD10 codes of comorbidities. Table S3. Association between CYP2C19 extensive and non‐extensive metabolizers and gastric cancer incidence (n = 199). Table S4. Associations of gastric cancer incidence with CYP2C19 extensive, intermediate, and poor metabolizers (n = 199). [file JGH3-5-770-s001.doc]

**Supplementary Figure 1.** **Cumulative incidence of gastric cancer according to (A) CYP2C19 extensive vs. non-extensive metabolizers and (B) CYP2C19 extensive vs. intermediate vs. poor metabolizers**


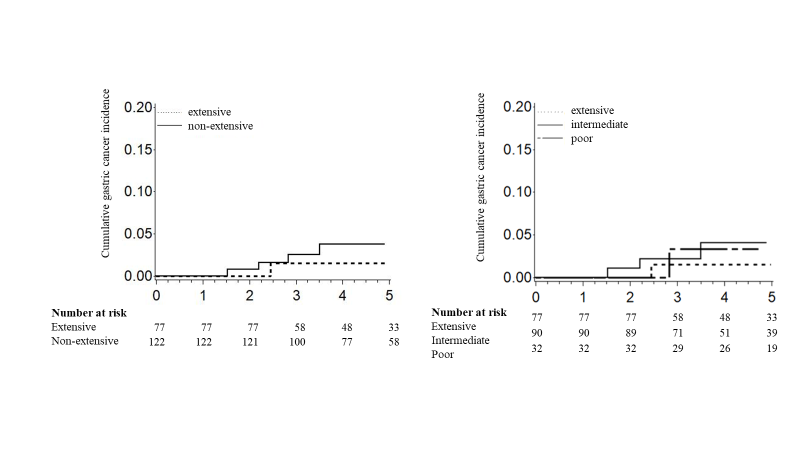


p=0.416 (log rank)

p=0.706 (log rank)

**(A)**

**(B)**

Survival analysis was performed using the Kaplan–Meier method and log-rank test.

**Supplementary Figure 2. Posterior probability of gastric cancer in CYP2C19 intermediate and poor metabolizers using a Bayesian model**


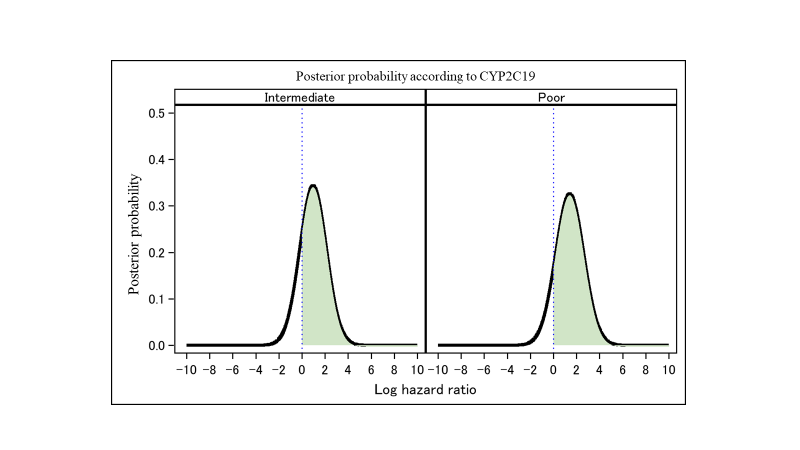


The blue area showed that the probability of the hazard ratio is greater than 1.

**Supplementary Figure 3. Cumulative incidence of gastric cancer in (A) PPI+NSAID high-dose and low-dose users and PPI-users, (B) PPI+NSAID long-term and short-term users and PPI-users.**


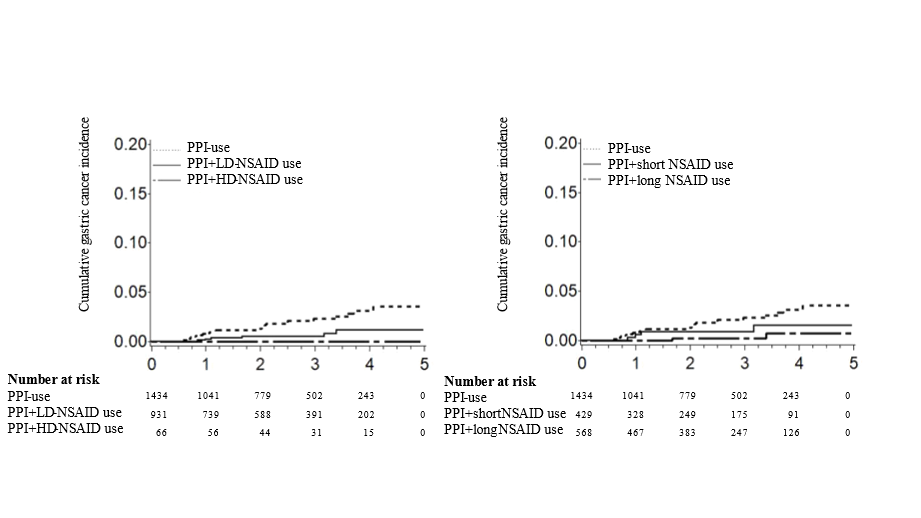


**(A)**

**(B)**

p=0.019* (log rank)

p=0.015* (log rank)

Survival analysis was performed using the Kaplan–Meier method and log-rank test.

Abbreviations: PPI, proton pump inhibitors; NSAIDs, nonsteroidal anti-inflammatory drugs; LD, low dose; HD, high dose..

**Supplementary Table 2. ICD10 codes of comorbidities.**

| Comorbidities | ICD-10 code |
| --- | --- |
| Atrial fibrillation | I480-I489 |
| AIDS | B200-B229, B24 |
| Arterial thrombosis | I740-I749 |
| Carotid disease | I652, I720 |
| Cerebrovascular disease | G450-G469, H340, I600-639, I64, I650-I699 |
| Chronic heart failure | I099, I110, I130, I132, I255, I420, I425-I439, I500-I509, P290 |
| Chronic kidney disease < stage 5 | I120, I131, N032-N037, N052-N057, N180-N189, N19, N250, Z490-Z492, Z940, Z992 |
| Chronic kidney disease stage 5 | N185 |
| Dementia | F000-F029, F03, F051, G300-G309, G311 |
| DM without complications | E100, E101, E106, E108-E111, E116, E118-E121, E126, E128-E131, E136, E138-E141, E146, E148, E149 |
| DM with complications | E102-E105, E107, E112-E117, E122-E125, E132-E135, E137, E142-E145,E147 |
| Deep vein thrombosis | I800-I809, I820-I829 |
| Hemiplegia | G041, G114, G801, G802, G810-G834, G839 |
| Hypertension | I10, I110-I159 |
| Dyslipidemia | E780-E785 |
| Ischemic heart disease | I210-I229, I252 |
| Liver disorder (mild) | B180-B189, K700-K703, K709, K713-K715, K717, K730-K749, K760, K762-K764, K768-K769, Z944 |
| Liver disorder (severe) | I850, I859, I864, I982, K704, K711, K721, K729, K765-K767 |
| Malignancy without metastasis | C000-C009, C01, C020-C69, C07, C080-C119, C12, C130-C189, C19, C20, C210-C229, C23, C240-C329, C33, C340-C349, C37, C380-C519, C52, C530-C549, C55,C56, C570-C570, C58, C600- C609, C61, C620-C639, C64, C65, C66, C670-C729, C73, C740-C769, C810-C969, C97 |
| Malignancy with metastasis | C770-C809 |
| Pulmonary embolism | I260-I269 |
| Peripheral vascular disease | I700-I719, I731, I738, I739, I771, I790, I792, K551, K558, K559, Z958, Z959 |
| Pulmonary disease | I278, I279, J40, J410—J419, J42, J430-J459, J46, J47, J60, J61, J620-J639, J64, J65, J660-J679, J684, J701, J703 |
| Rheumatic disease | M050-M069, M315, M320-M349, M351, M353, M360 |
| Transient ischemic attack | G459 |
| Peptic ulcer disease | K250-K289 |
| Unstable angina disease | I200-I209 |
| Valvular disease | I340-I379 |

**Supplementary Table 3. Association between CYP2C19 extensive and non-extensive metabolizers and gastric cancer incidence (n = 199).**

| **Factor** | **Gastric cancer, n = 5** | **Non-gastric cancer, n = 194** | **Crude HR**  **(95% CI)** | **Adjusted HR**†  **(95% CI)** | **p value** |
| --- | --- | --- | --- | --- | --- |
| CYP2C19 |  |  |  |  |  |
| Extensive | 1 | 76 | 1 | 1 |  |
| Non-extensive | 4 | 118 | 2.41 (0.27 to 21.59) | 2.47 (0.27 to 22.52) | 0.423 |

Abbreviations: HR, hazard ratio; CI, confidence interval

†HR adjusted for age > 70 years and sex.

**Supplementary Table 4. Associations of gastric cancer incidence with CYP2C19 extensive, intermediate, and poor metabolizers (n = 199).**

| **Factor** | **Gastric cancer, n = 5** | **Non-gastric cancer, n = 194** | **Crude HR (95% CI)** | **Adjusted HR**†  **(95% CI)** | **p value** |
| --- | --- | --- | --- | --- | --- |
| CYP2C19 |  |  |  |  |  |
| Extensive | 1 | 76 | 1 | 1 |  |
| Intermediate | 3 | 87 | 2.54 (0.26 to 24.43) | 2.63 (0.27 to 25.84) | 0.408 |
| Poor | 1 | 31 | 2.10 (0.13 to 33.58) | 2.11 (0.13 to 33.96) | 0.600 |

Abbreviations: HR, hazard ratio; CI, confidence interval

†HR adjusted for age (> 70 years) and sex.
