# Supplementary material for: Nonsteroidal anti‐inflammatory drugs prevent gastric cancer associated with the use of proton pump inhibitors after Helicobacter pylori eradication
Source: JGH Open. 2021 Jun 5;5(7):770–7. doi: 10.1002/jgh3.12583 (PMC8264245; doi:10.1002/jgh3.12583)
Supplement: Supplementary file 2 — Table S1. Drug codes. [file JGH3-5-770-s002.docx]

**Supplementary Table1. Drug codes.**

| Combination agents of pylori eradication |
| --- |
| 610462048 |
| 610462049 |
| 622029101 |
| 622289101 |
| 622289201 |
| 622289301 |
| 622485401 |
| 622485501 |
| 622485601 |
| Amoxicillin |
| 620006829 |
| 620008584 |
| 620009117 |
| 621073901 |
| 622745400 |
| 616130040 |
| 616130132 |
| 620006919 |
| 616130295 |
| 620007025 |
| Clarithromycin |
| 616140105 |
| 620003926 |
| 620003927 |
| 620003928 |
| 620003929 |
| 620003930 |
| 620003931 |
| 620003932 |
| 620003933 |
| 620003934 |
| 620003935 |
| 620003939 |
| 620006670 |
| 620008013 |
| 621736701 |
| 621742103 |
| 621752901 |
| 622079401 |
| 622659101 |
| 622746200 |
| 616140102 |
| Metronidazole |
| 620007057 |
| Proton Pump Inhibitors |
| 610412202 |
| 610412203 |
| 610443068 |
| 610443069 |
| 610443070 |
| 610443071 |
| 610462010 |
| 610462011 |
| 612320549 |
| 612320550 |
| 620001983 |
| 620001984 |
| 620001985 |
| 620001986 |
| 620002694 |
| 620002695 |
| 620002743 |
| 620002744 |
| 620002749 |
| 620002750 |
| 620002871 |
| 620002872 |
| 620003914 |
| 620004087 |
| 620004088 |
| 620005581 |
| 620005583 |
| 620005584 |
| 620005585 |
| 620007127 |
| 620007128 |
| 620009451 |
| 620009452 |
| 621622303 |
| 621622403 |
| 621630104 |
| 621630502 |
| 621630601 |
| 621630701 |
| 621673701 |
| 621673801 |
| 621680901 |
| 621681001 |
| 621681401 |
| 621693101 |
| 621693201 |
| 621743701 |
| 621743801 |
| 621780301 |
| 621780303 |
| 621780401 |
| 621780403 |
| 621794301 |
| 621796301 |
| 621796401 |
| 621919001 |
| 621919101 |
| 621977902 |
| 621983103 |
| 621997201 |
| 621997301 |
| 621999501 |
| 621999601 |
| 621999901 |
| 622000001 |
| 622001201 |
| 622001301 |
| 622002201 |
| 622002301 |
| 622005301 |
| 622005401 |
| 622007301 |
| 622007401 |
| 622011201 |
| 622011301 |
| 622012901 |
| 622013001 |
| 622015801 |
| 622015901 |
| 622016201 |
| 622016301 |
| 622020602 |
| 622020702 |
| 622021401 |
| 622021501 |
| 622023101 |
| 622023201 |
| 622023301 |
| 622023401 |
| 622025601 |
| 622025701 |
| 622026001 |
| 622026101 |
| 622031601 |
| 622031701 |
| 622032801 |
| 622032901 |
| 622034601 |
| 622034701 |
| 622035501 |
| 622035601 |
| 622040101 |
| 622040201 |
| 622060201 |
| 622060301 |
| 622077801 |
| 622080701 |
| 622080801 |
| 622089501 |
| 622089601 |
| 622118601 |
| 622118701 |
| 622402601 |
| 622469801 |
| 622469901 |
| 622505501 |
| 622505601 |
| 622617000 |
| 622617100 |
| 622617200 |
| 622624801 |
| 622624901 |
| Potassium Competitive Acid Blockers |
| 622404401 |
| 622404501 |
| Non-Steroidal Anti-Inflammatory Drugs |
| 610406382 |
| 610406383 |
| 610406384 |
| 610406387 |
| 610406388 |
| 610406402 |
| 610422322 |
| 610433119 |
| 610443079 |
| 610443080 |
| 610454052 |
| 610463033 |
| 610463034 |
| 610463037 |
| 610463150 |
| 611140098 |
| 611140138 |
| 611140139 |
| 611140236 |
| 611140237 |
| 611140322 |
| 611140323 |
| 611140395 |
| 611140431 |
| 611140435 |
| 611140828 |
| 611140844 |
| 611140845 |
| 611140846 |
| 611140847 |
| 620002043 |
| 620002057 |
| 620002431 |
| 620002432 |
| 620002516 |
| 620002531 |
| 620002537 |
| 620002646 |
| 620002647 |
| 620003153 |
| 620003154 |
| 620003523 |
| 620003524 |
| 620003624 |
| 620004494 |
| 620004626 |
| 620004857 |
| 620004858 |
| 620004916 |
| 620004917 |
| 620006095 |
| 620006174 |
| 620006848 |
| 620006849 |
| 620006859 |
| 620007059 |
| 620007068 |
| 620007095 |
| 620007096 |
| 620007098 |
| 620007099 |
| 620007100 |
| 620007129 |
| 620007150 |
| 620007151 |
| 620007152 |
| 620007153 |
| 620008114 |
| 620008115 |
| 620008117 |
| 620008118 |
| 620008119 |
| 620008120 |
| 620008121 |
| 620008122 |
| 620008123 |
| 620008124 |
| 620008125 |
| 620008126 |
| 620008127 |
| 620008128 |
| 620008129 |
| 620008130 |
| 620008131 |
| 620008132 |
| 620008133 |
| 620008135 |
| 620008136 |
| 620008137 |
| 620008138 |
| 620008139 |
| 620008140 |
| 620008141 |
| 620008142 |
| 620008143 |
| 620008144 |
| 620008145 |
| 620008146 |
| 620008147 |
| 620008148 |
| 620008149 |
| 620008150 |
| 620008151 |
| 620008625 |
| 620008628 |
| 620008632 |
| 620008646 |
| 620008780 |
| 620079303 |
| 620079305 |
| 620079311 |
| 620079315 |
| 620079325 |
| 620079338 |
| 620079345 |
| 620081301 |
| 620088902 |
| 620090601 |
| 620094401 |
| 620097508 |
| 620097815 |
| 620098401 |
| 620098501 |
| 620098702 |
| 620098801 |
| 620098902 |
| 620099003 |
| 620099101 |
| 620099201 |
| 620099301 |
| 620099501 |
| 620099601 |
| 620099701 |
| 620100001 |
| 620100501 |
| 620100602 |
| 620100702 |
| 620100901 |
| 621212601 |
| 621215101 |
| 621215401 |
| 621215602 |
| 621392002 |
| 621466002 |
| 621466202 |
| 621466401 |
| 621466601 |
| 621534501 |
| 621623201 |
| 621634301 |
| 621635802 |
| 621640201 |
| 621640501 |
| 621808201 |
| 621837703 |
| 621837803 |
| 621936001 |
| 621981502 |
| 622011102 |
| 622012401 |
| 622014601 |
| 622022501 |
| 622034902 |
| 622051201 |
| 622058201 |
| 622062601 |
| 622066701 |
| 622314000 |
| 622325600 |
| Aspirin |
| 610443053 |
| 611140017 |
| 611140798 |
| 611140849 |
| 611140850 |
| 620000065 |
| 620000484 |
| 620001952 |
| 620004280 |
| 620007816 |
| 620008577 |
| 620009301 |
| 620072734 |
| 621362001 |
| 621374801 |
| 621374901 |
| 621375001 |
| 621391201 |
| 621419201 |
| 621419401 |
| 621675501 |
| 621676502 |
| 622258001 |
| Statin |
| 610443013 |
| 610443014 |
| 610454084 |
| 610454085 |
| 610462015 |
| 610462016 |
| 610470012 |
| 610470013 |
| 610470014 |
| 612180263 |
| 612180264 |
| 612180265 |
| 620000052 |
| 620000053 |
| 620000103 |
| 620000104 |
| 620000105 |
| 620000106 |
| 620000107 |
| 620000108 |
| 620000159 |
| 620000160 |
| 620000176 |
| 620000422 |
| 620000423 |
| 620002477 |
| 620002478 |
| 620002736 |
| 620002798 |
| 620002799 |
| 620002800 |
| 620004038 |
| 620008053 |
| 620008054 |
| 620008055 |
| 620008056 |
| 620009322 |
| 620009323 |
| 620009324 |
| 620009325 |
| 621521301 |
| 621521401 |
| 621523101 |
| 621523201 |
| 621524102 |
| 621524402 |
| 621525701 |
| 621525801 |
| 621528602 |
| 621528702 |
| 621528801 |
| 621528901 |
| 621529001 |
| 621529101 |
| 621531001 |
| 621531101 |
| 621531703 |
| 621532501 |
| 621532601 |
| 621532902 |
| 621533002 |
| 621533101 |
| 621533201 |
| 621533501 |
| 621533601 |
| 621533801 |
| 621533901 |
| 621534003 |
| 621534101 |
| 621534204 |
| 621534301 |
| 621623603 |
| 621635202 |
| 621639001 |
| 621639101 |
| 621639701 |
| 621639801 |
| 621643301 |
| 621643401 |
| 621643501 |
| 621643601 |
| 621675101 |
| 621694001 |
| 621752501 |
| 621934801 |
| 621934901 |
| 621935001 |
| 621948701 |
| 621948801 |
| 621955003 |
| 621964101 |
| 621964201 |
| 621964301 |
| 621964401 |
| 621964501 |
| 621964601 |
| 621981403 |
| 622015101 |
| 622015201 |
| 622015301 |
| 622052801 |
| 622055602 |
| 622071601 |
| 622075801 |
| 622075901 |
| 622076401 |
| 622076501 |
| 622098401 |
| 622098501 |
| 622099101 |
| 622099201 |
| 622102502 |
| 622107601 |
| 622107701 |
| 622110401 |
| 622110501 |
| 622116802 |
| 622116902 |
| 622126901 |
| 622127001 |
| 622128201 |
| 622128301 |
| 622136401 |
| 622139600 |
| 622143801 |
| 622143901 |
| 622152001 |
| 622152101 |
| 622161801 |
| 622161901 |
| 622165601 |
| 622165701 |
| 622167601 |
| 622167701 |
| 622169902 |
| 622170002 |
| 622170101 |
| 622170201 |
| 622180602 |
| 622180702 |
| 622186601 |
| 622186701 |
| 622187601 |
| 622187701 |
| 622204801 |
| 622204901 |
| 622217101 |
| 622217201 |
| 622239201 |
| 622239301 |
| 622241301 |
| 622241401 |
| 622244801 |
| 622244901 |
| 622252001 |
| 622252101 |
| 622268001 |
| 622268101 |
| 622268201 |
| 622269101 |
| 622269201 |
| 622270001 |
| 622270101 |
| 622271801 |
| 622271901 |
| 622273101 |
| 622273201 |
| 622273301 |
| 622274901 |
| 622275001 |
| 622275101 |
| 622276301 |
| 622276401 |
| 622276501 |
| 622280201 |
| 622280301 |
| 622280401 |
| 622280501 |
| 622280601 |
| 622280701 |
| 622280801 |
| 622282201 |
| 622282301 |
| 622283701 |
| 622283801 |
| 622285001 |
| 622285101 |
| 622286201 |
| 622286301 |
| 622286401 |
| 622287601 |
| 622289501 |
| 622289601 |
| 622291801 |
| 622291901 |
| 622292001 |
| 622292301 |
| 622292401 |
| 622292501 |
| 622293301 |
| 622293401 |
| 622294301 |
| 622294401 |
| 622294501 |
| 622296001 |
| 622296101 |
| 622296201 |
| 622297101 |
| 622297201 |
| 622298001 |
| 622298101 |
| 622298201 |
| 622299001 |
| 622299101 |
| 622302401 |
| 622302501 |
| 622302801 |
| 622302901 |
| 622304601 |
| 622304701 |
| 622304801 |
| 622304901 |
| 622315400 |
| 622315500 |
| 622321900 |
| 622342801 |
| 622347401 |
| 622359101 |
| 622360101 |
| 622362701 |
| 622365801 |
| 622372401 |
| 622387601 |
| 622392501 |
| 622406901 |
| 622419701 |
| 622419801 |
| 622419901 |
| 622421601 |
| 622421701 |
| 622421801 |
| 622426201 |
| 622427701 |
| 622427801 |
| 622431401 |
| 622434301 |
| 622434401 |
| 622441101 |
| 622441201 |
| 622457701 |
| 622457801 |
| 622457901 |
| 622464901 |
| 622465001 |
| 622465101 |
| 622475000 |
| 622475100 |
| 622512001 |
| 622512101 |
| 622512201 |
| 622522101 |
| 622522201 |
| 622522301 |
| 622524501 |
| 622524601 |
| 622524701 |
| 622528901 |
| 622529001 |
| 622537301 |
| 622537401 |
| 622568601 |
| 622571801 |
| 622571901 |
| 622572801 |
| 622572901 |
| 622575201 |
| 622575301 |
| 622575401 |
| 622575501 |
| 622575601 |
| 622575701 |
| 622577901 |
| 622578001 |
| 622578101 |
| 622578201 |
| 622578401 |
| 622578501 |
| 622578601 |
| 622578701 |
| 622578801 |
| 622581601 |
| 622581701 |
| 622581801 |
| 622581901 |
| 622582001 |
| 622582101 |
| 622582501 |
| 622582601 |
| 622582701 |
| 622582801 |
| 622584201 |
| 622584701 |
| 622584801 |
| 622586001 |
| 622586101 |
| 622586201 |
| 622586301 |
| 622588801 |
| 622588901 |
| 622589001 |
| 622589101 |
| 622590101 |
| 622590201 |
| 622590301 |
| 622590401 |
| 622591701 |
| 622591801 |
| 622591901 |
| 622592001 |
| 622592601 |
| 622592701 |
| 622592901 |
| 622593001 |
| 622593101 |
| 622593201 |
| 622595301 |
| 622595401 |
| 622598301 |
| 622598401 |
| 622598501 |
| 622598601 |
| 622599201 |
| 622599301 |
| 622599401 |
| 622599501 |
| 622600301 |
| 622600601 |
| 622600701 |
| 622600801 |
| 622600901 |
| 622601201 |
| 622601301 |
| 622601401 |
| 622601501 |
| 622604001 |
| 622604101 |
| 622604201 |
| 622605001 |
| 622605101 |
| 622605201 |
| 622605301 |
| 622605401 |
| 622606601 |
| 622606701 |
| 622615600 |
| 622615700 |
| 622615800 |
| 622615900 |
| 622640801 |
| 622640901 |
| 622644801 |
| 622644901 |
| 622660001 |
| 622660101 |
| 622665901 |
| 622666001 |
| 622666101 |
| 622676701 |
| 622676801 |
| 622691000 |
| 622691800 |
| 622692400 |
| 622692500 |
| 622692600 |
| 622692700 |
| Fibrate |
| 610407028 |
| 610422262 |
| 610422263 |
| 610422264 |
| 610422265 |
| 610422276 |
| 612180028 |
| 612180029 |
| 612180106 |
| 620002123 |
| 620008508 |
| 620338317 |
| 620339201 |
| 620339401 |
| 620339501 |
| 620340201 |
| 620340603 |
| 620340901 |
| 620341001 |
| 620341301 |
| 621254601 |
| 622026702 |
| 622039501 |
| 622039601 |
| 622090701 |
| 622090801 |
| 622096102 |
| 622096801 |
| 622096901 |
| 622223601 |
| 622573101 |
| 622590501 |
| 622590601 |
| Other lipid lowering |
| 610432003 |
| 610462007 |
| 610463087 |
| 612180004 |
| 612180140 |
| 612180141 |
| 612180292 |
| 620002508 |
| 620003669 |
| 620004459 |
| 620004868 |
| 620005785 |
| 620005920 |
| 620006115 |
| 620006870 |
| 620008631 |
| 620346008 |
| 620346018 |
| 620346023 |
| 620346029 |
| 620346101 |
| 622198801 |
| 622516701 |
| 622516801 |
| 622516901 |
| 622584201 |
| 622584701 |
| 622676701 |
| 622676801 |
| Metformin |
| 620004480 |
| 620005570 |
| 621676001 |
| 621974701 |
| 621986301 |
| 621986401 |
| 622242501 |
| 622412701 |
| 622417101 |
| 622417201 |
| 622421101 |
| 622421201 |
| 622421901 |
| 622422001 |
| 622424401 |
| 622424501 |
| 622427201 |
| 622427301 |
| 622432601 |
| 622432701 |
| 622436301 |
| 622438401 |
| 622438501 |
| 622448601 |
| 622450301 |
| 622450401 |
| 622466601 |
| 622517101 |
| 622654401 |
| 622654501 |
